# Genome-Wide Identification and Comprehensive Analysis of Ubiquitin-Specific Protease Gene Family in Soybean (*Glycine max*)

**DOI:** 10.3390/ijms26146689

**Published:** 2025-07-11

**Authors:** Cuirong Tan, Dingyue Ban, Haiyang Li, Jinxing Wang, Baohui Liu, Chunyu Zhang

**Affiliations:** 1 Guangdong Provincial Key Laboratory of Plant Adaptation and Molecular Design, Innovative Center of Molecular Genetics and Evolution, School of Life Sciences, Guangzhou University, Guangzhou 510006, China; cuirongt@foxmail.com (C.T.); lihy@gzhu.edu.cn (H.L.); 2 Guangdong Provincial Key Laboratory of Applied Botany & State Key Laboratory of Plant Diversity and Specialty Crops, South China Botanical Garden, Chinese Academy of Sciences, Guangzhou 510650, China; bandingyue22@mails.ucas.ac.cn; 3 Suihua Branch of Heilongjiang Academy of Agricultural Sciences, Suihua 152052, China; wjxsuihua@126.com

**Keywords:** soybean, ubiquitin-specific proteases (UBPs), haplotype analysis, gene expression

## Abstract

Deubiquitination plays a pivotal role in regulating plant responses to abiotic stress, growth, and development. Among the deubiquitinase (DUB) families, ubiquitin-specific proteases (UBPs) constitute the largest group. Despite this, limited research has been conducted on the functional characteristics of the *UBP* gene family in soybean (*Glycine max*). In this study, we identified 52 *UBP* gene family members in soybean, all of which harbored UCH (ubiquitin C-terminal hydrolase) domains with short yet evolutionarily conserved Cys-box and His-box. These genes were phylogenetically classified into 14 distinct groups; *GmUBP* genes within the same group shared analogous patterns of conserved domains and motifs. Moreover, a synteny analysis reveals that the *GmUBP* family has undergone extensive gene duplication events and shares a close evolutionary relationship with *Arabidopsis thaliana*. We conducted a focused analysis on *GmUBP7*, which is a gene exhibiting high expression levels in soybean seeds. Intriguingly, this gene exhibited several haplotypes in natural soybean varieties, with significant differences being observed in relation to seed traits, such as 100-seed weight, total fatty acid content, and protein content among different haplotypes. Collectively, the findings from this study provide a foundation for the functional characterization of *GmUBP* genes, offering new insights into the regulatory network underlying seed development in soybean.

## 1. Introduction

Soybean (*Glycine max* (L.) Merr.) is a globally significant crop that is utilized as an oil, feed, and forage source; it is a major provider of edible oil and plant protein for humans and livestock worldwide [1]. Yield and quality in soybean plants are closely tied to seed development, which is regulated by multiple signaling pathways, including the ubiquitin–proteasome pathway, the G-protein signaling pathway, the mitogen-activated protein kinase (MAPK) signaling pathway, plant hormone pathways, the HAIKU (IKU) pathway, and transcription regulatory factors [2]. Among these pathways, ubiquitination stands out as a critical process, influencing plant growth and development by regulating the activity and stability of target proteins [3].

Protein ubiquitination and deubiquitination are well-established fields of study [4,5]. The ubiquitination process involves three sequential steps and is mediated by a cascade of the following three enzymes: the E1 ubiquitin-activating enzyme, the E2 ubiquitin-conjugating enzyme, and the E3 ubiquitin ligase [3,6]. Deubiquitination, which is the opposite process of ubiquitination, involves deubiquitinating enzymes (DUBs) and removing ubiquitin (Ub) from ubiquitinated proteins in order to stabilize them [7]. Notably, in plants, ubiquitin-specific proteases (UBPs) form the largest and most diverse subfamily of DUBs. UBPs are involved in two primary biochemical activities: cleaving linked ubiquitin chains from proteins conjugated with ubiquitin and producing mature ubiquitin from its precursor [3]. All UBPs possess a ubiquitin C-terminal hydrolase (UCH) domain—this is unique to UBP proteins and is known as the UBP domain—along with two short but highly conserved motifs: the cysteine (Cys) box and the histidine (His) box. These motifs contain key catalytic residues—the cysteine in the Cys-box and histidine and aspartic acid/asparagine in the His-box—which are essential for the deubiquitination activity of UBPs [8,9].

The ubiquitin-specific peptidase (UBP) family has been characterized in various plant species, exhibiting marked interspecific differences in gene numbers. Specifically, genome-wide analyses have revealed 27 *UBP* genes in *Arabidopsis thaliana* [10], 25 in *Oryza sativa* [11], 48 in *Phyllostachys edulis* [12], 97 in *Triticum aestivum* [13], and 45 in *Zea mays* [14]. These enzymes play critical roles in diverse stress response pathways, including abscisic acid (ABA)-mediated drought tolerance, salt stress adaptation, and immune responses, as well as other key biological processes [15,16,17,18,19]. In Arabidopsis, the overexpression of *UBP12*/*UBP13* enhances ORESARA1 (ORE1) protein levels, thereby promoting nitrogen-deficiency-induced leaf senescence [20]. Additionally, UBP12 and UBP13 regulate plant growth responses under nitrogen starvation and post-carbon starvation recovery by stabilizing the BRI1 EMS SUPPRESSOR 1 (BES1) transcription factor. Furthermore, these enzymes counteract root meristem growth factor 1 (RGF1)-induced ubiquitination by interacting directly with RGF1 receptors, thereby promoting root meristem development [21]. Interestingly, *UBP12* and *UBP13* also contribute to the regulation of flowering time and circadian rhythms [22]. Loss-of-function mutations in *AtUBP1* and *AtUBP2* result in hypersensitivity to the amino acid analog canavanine (CAN), accompanied by severe developmental defects, including dwarfism, short root growth, and chlorosis [10]. During reproductive growth, *UBP15* has been shown to regulate seed development in both Arabidopsis and rice. It antagonizes DA1-mediated ubiquitin receptor signaling to modulate organ development and seed size while promoting maternal bead tissue proliferation to enhance seed size [23]. In rice, OsUBP15 interacts directly with OsDA1 to regulate grain dimensions [24]. Other *UBP* gene family members also contribute uniquely to plant reproduction, e.g., *AtUBP3* and *AtUBP4* are essential for male gametophyte development [25], *AtUBP14* participates in early embryogenesis [26], and *AtUBP26* plays a role in histone ubiquitination and seed development [27,28]. Moreover, *OsUBP2* has been identified as a negative regulator of reactive oxygen species (ROS) accumulation and immune responses in rice [19]. Subsequent studies have demonstrated that OsUBP2 modulates rice cell death and immune responses by stabilizing the SPOTTED LEAF 35 (SPL35) protein [29]. Furthermore, this protein plays a role in rice immunity by deubiquitinating H2B; this is a process that regulates genes in the salicylic acid (SA) biosynthesis pathway [30]. In addition, rice seedlings with a homozygous T-DNA insertion mutant in *OsUBP6* exhibit defective phenotypes during early growth, though these defects diminish as the plants mature [31]. In maize, the homologs of *AtUBP16*, namely, *ZmUBP15*, *ZmUBP16*, and *ZmUBP19*, are essential for the plant’s response to salt and cadmium stress [32].

In soybean, the *UBP* gene family remains poorly characterized compared to other plant species. To date, only one *GmUBP* gene has been comprehensively studied, encoding a ubiquitin-specific protease orthologous to *AtUBP22*. This gene, designated as *GmSW17* (*Seed Width 17*), has been shown to determine seed width and weight in natural soybean populations. Population genomics analyses reveal that *GmSW17* underwent artificial selection during soybean domestication but has not been fully fixed in modern cultivars [33]. To investigate the roles of soybean *UBP* genes in plant growth and development, we systematically identified 52 *GmUBP* genes across the soybean genome. Subsequent analyses focused on their conserved motifs, gene structures, chromosomal distributions, and expression patterns. To elucidate their evolutionary relationships with *UBP* genes in other plant species, phylogenetic tree construction and collinearity analysis were performed. Additionally, we conducted an in-depth investigation of *GmUBP7*, analyzing its expression levels across multiple stages of soybean seed development using RT-qPCR. Haplotype analysis further reveals significant differences in 100-seed weight, total fatty acid content, and protein content among distinct haplotypes. The findings of this study contribute to a better understanding of the functional roles of *GmUBP* genes in seed development and provide a foundation for identifying the roles of this essential gene family in soybean agronomics and improvement.

## 2. Results

### 2.1. Identification and Characterization of the UBP Genes in Soybean

To identify members of the *UBP* gene family in soybean, we initially queried the relevant databases using 27 Arabidopsis UBP protein sequences. This analysis yielded 63 putative *GmUBP* genes, which were further validated by examining their conserved UCH domain using the Pfam database and NCBI CD Search. After removing non-conforming sequences, 52 *GmUBP* genes were identified in the soybean genome and were systematically named *GmUBP1* to *GmUBP52* based on their chromosomal locations (Table S1). All 52 GmUBPs contained the UCH domain.

Detailed information about these genes, including gene length, physical and chemical properties, transmembrane domains, and subcellular localization, is presented in Table S1. The coding sequences of the *GmUBP* genes ranged from 1107 to 3423 bp, while their protein lengths spanned 369–1141 amino acids. The molecular weight (MW) of these proteins varied between 42.052 and 133.168 kDa, with isoelectric points (pIs) ranging from 4.96 to 9.2. Predictions for transmembrane domains reveal that 11 GmUBPs contained a single TM domain (e.g., GmUBP2). Subcellular localization analyses provided insights into the functional roles of these proteins. The results indicate that 24 GmUBPs were localized in the nucleus, while 13 were found in the plasma membrane. Additionally, seven GmUBPs were associated with the endomembrane system, and four were localized in chloroplasts. Notably, GmUBP5 (Glyma.02G213400) and GmUBP11 (Glyma.04G091700) were predicted to reside in the extracellular space, whereas GmUBP24 (Glyma.09G225200) and GmUBP29 (Glyma.12G011800) were localized in organelle membranes. Finally, all 52 GmUBPs exhibited hydrophilicity, as their predicted hydrophobicity scores were less than zero (Table S1).

### 2.2. Gene Structures, Conserved Motifs, and cis-Acting Element Analysis of the GmUBP Genes

**Figure 1:**
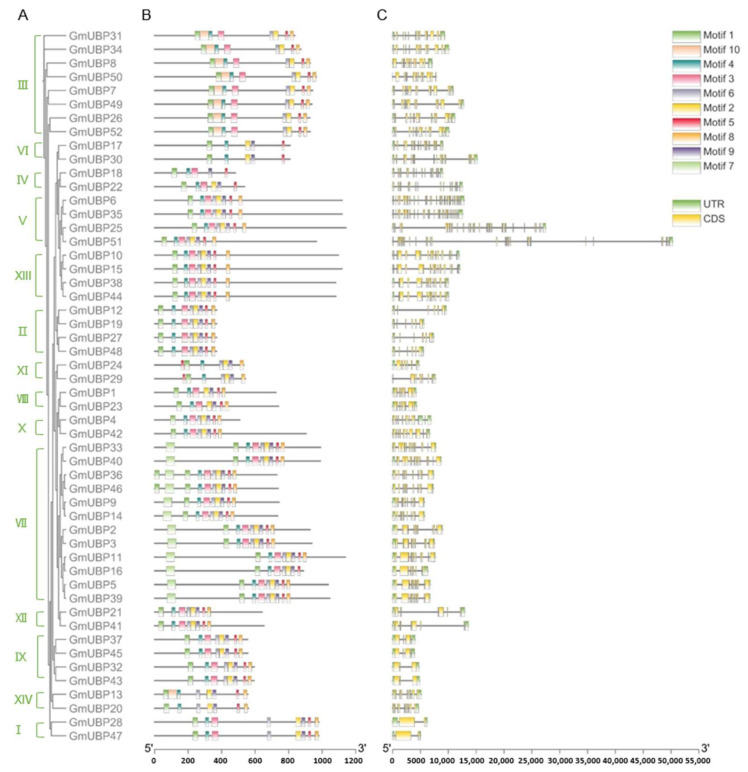
The gene and protein structures of 52 *GmUBP* genes. (**A**): The evolution tree of the *UBP* genes from *Glycine max*. (**B**): Ten conserved motifs were identified using MEME, with different color boxes representing distinct motifs. (**C**): The intron/exon structure of *UBP* genes was analyzed via WebLogo 3, where the yellow box denotes exons, the gray line signifies introns, and the green box represents untranslated regions (UTRs). The different subfamilies are labeled from I to XIV.

In order to elucidate the relationship between gene function and evolution, we analyzed the structural organization and conserved motifs of *GmUBP* genes (Table S2–S4). The *GmUBP25* gene (V subfamily) had the largest number of exons (32), while *GmUBP28* and *GmUBP47* (I subfamily) had the smallest number of exons (2). Other *GmUBP* genes contained exon numbers ranging from 3 to 31. Genes within the same subfamily exhibited similar intron–exon structures, suggesting functional conservation within the soybean *UBP* gene family. The variability in exon numbers among *GmUBP* genes may indicate functional diversification within the family (Figure 1A,C).

To further investigate the evolutionary changes in the *GmUBP* family, we analyzed the conserved motifs in the 52 GmUBP proteins using MEME online software, identifying 10 distinct motifs (designated motifs 1–10). Motifs 1, 4, and 5 were consistently present in all GmUBP proteins, and their order (1, 4, and 5) was consistent except for GmUBP24 and GmUBP29. Additionally, motif 10 was primarily found in subfamily III, while motif 7 was exclusive to subfamily VII (Figure 1B, Table S5).

**Figure 2:**
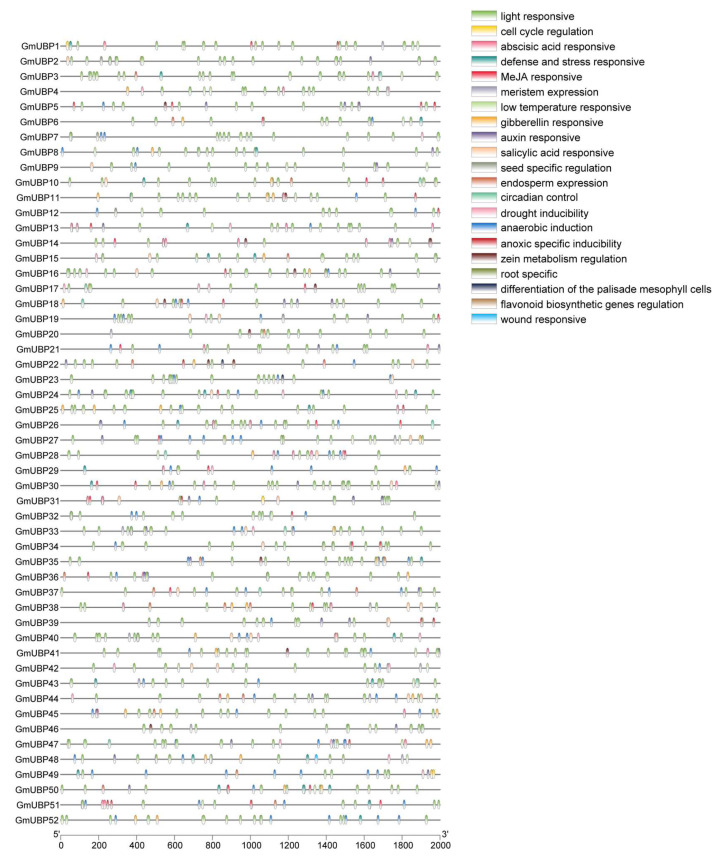
The Cis-acting element analysis of 52 *GmUBP* gene promoters. The *GmUBP* genes were classified according to their phylogenetic relationships. The response elements are indicated by 21 differently colored columns, where each column represents a distinct element type. The horizontal coordinates correspond to gene length.

For promoter analysis, we directly extracted promoter sequences (2000 bp upstream of the start codon—ATG) for the 52 *GmUBP* genes from the soybean genome sequence. Using the PlantCare online tool, we predicted cis-acting elements, which were subsequently visualized using TBtools software. These elements included hormone-responsive motifs (e.g., ABA, IAA, GA, MeJA, and SA), environmental stress response elements (e.g., low temperature, light responses, and circadian rhythms), and anaerobic induction elements. Of these, light-responsive elements and anaerobic induction elements were the most prevalent (Figure 2).

### 2.3. Visualization of Chromosomal Location and Duplication of GmUBPs

**Figure 3:**
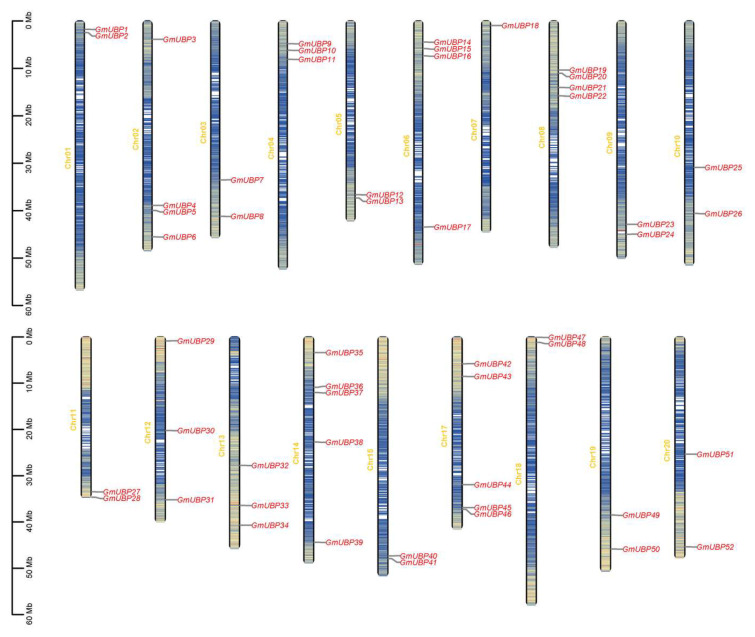
The chromosome distribution of *UBP* genes in *Glycine max*. The visualization shows the distribution of *UBP* genes in *Glycine max*. Chromosome names are labeled above each corresponding bar, while the vertical scale on the left indicates chromosome sizes in million base pairs (Mb). The 52 *GmUBP* genes are highlighted in red and the yellow to blue gradient represents different gene density, with yellow as high gene density region and blue as low gene density region.

To examine the chromosomal distribution of *GmUBP* genes, their chromosomal locations were determined. The results reveal that the 52 *GmUBP* genes were mapped to 20 chromosomes, with an uneven distribution across each chromosome (Figure 3). Specifically, chromosomes 14 and 17 each contained five *GmUBP* genes, while chromosomes 2, 6, and 8 each had four *GmUBP* genes. Chromosomes 4, 12, and 13 each harbored three *GmUBP* genes. Chromosomes 1, 3, 5, 9, 10, 11, 15, 18, 19, and 20 each carried two *GmUBP* genes. Notably, only one *GmUBP* gene was found on chromosome 7. Additionally, there was no apparent correlation between the number of *GmUBP* genes and the soybean chromosome length.

**Figure 4:**
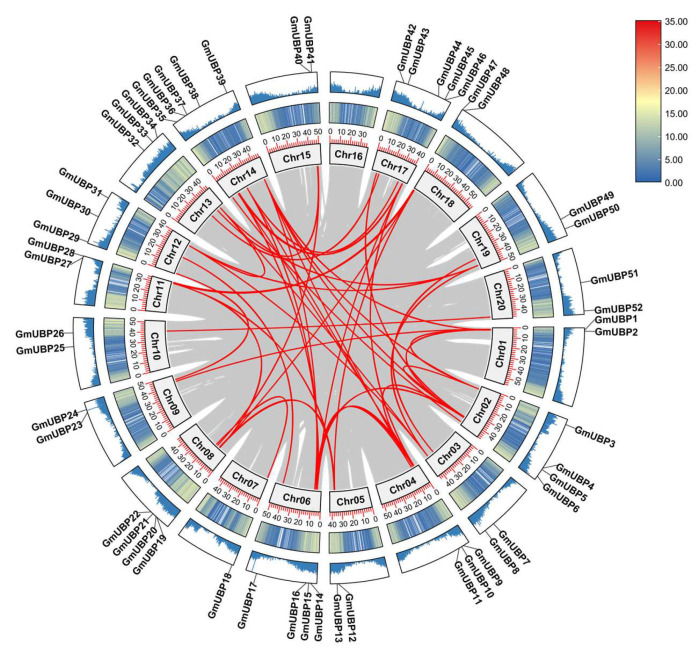
The duplication of 52 *GmUBP* genes during evolution. Chr1-Chr20, representing the 20 soybean chromosomes, are shown in the innermost circle. The range 0–35 indicates gene distances along the chromosomes. The blue column illustrates the density of gene distribution across soybean chromosomes, while the outermost circle shows the distribution of the 52 *GmUBP* genes. Gray lines depict genome-wide gene duplications during soybean evolution, whereas red lines specifically represent duplications of the *GmUBP* genes.

Furthermore, the gene duplication events of the *GmUBP* family within the soybean genome were analyzed (Figure 4). Gene duplication can occur through mechanisms such as tandem duplication and segmental duplication [34]. In total, 47 gene pairs undergoing segmental duplication were identified across 19 chromosomes (except for chr16), with no evidence of tandem duplications. This suggests that segmental duplications may play a dominant role in the expansion of *GmUBP* genes.

### 2.4. Phylogenetic Analysis and Collinearity Analysis of the UBP Genes Among Arabidopsis, Soybean, and Rice

**Figure 5:**
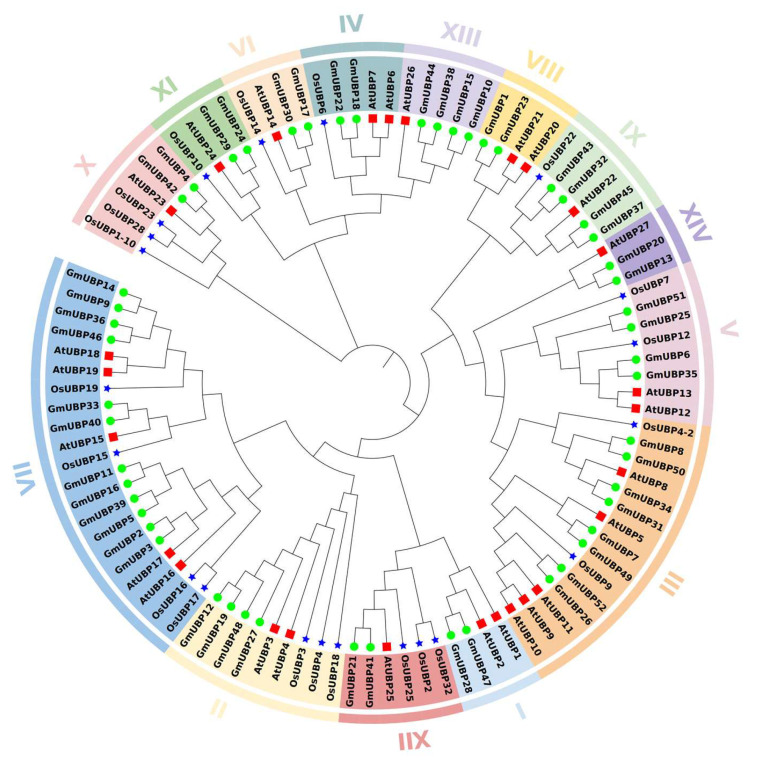
The phylogenetic relationships of UBPs from Arabidopsis, soybean, and rice. The phylogenetic tree was constructed using the full-length amino acid sequences of UBPs from three plant species: *Arabidopsis thaliana* (At), *Oryza sativa* (Os), and *Glycine max* (Gm). In this tree, green circles represent *Glycine max*, blue stars represent *Oryza sativa*, and the red box indicates *Arabidopsis thaliana*. The different subfamilies are labeled from I to XIV.

To investigate the phylogenetic organization of the UBP family, we conducted a comprehensive phylogenetic analysis using the protein sequences of 52 soybean UBPs, 32 rice UBPs, and 27 Arabidopsis UBPs (Tables S6 and S7). The phylogenetic tree was constructed using the neighbor-joining (NJ) method, followed by Maximum Likelihood (ML) analysis. Based on their phylogenetic relationships, the UBPs were classified into 14 distinct groups, designated as I to XIV (Figure 5). Notably, Group VII exhibited the largest membership, comprising 12 GmUBP proteins, while Group III contained 8 GmUBP proteins, representing the second-largest group. Interestingly, many UBP genes in soybean exist as closely related pairs, such as GmUBP4 and GmUBP42, further supporting the presence of paralogous genes within the soybean UBP family.

However, no orthologous relationships were observed among the UBP genes of soybean, rice, and Arabidopsis, suggesting that these UBP genes have undergone functional divergence during evolution. Despite this divergence, UBP proteins from soybean and Arabidopsis were distributed across 15 phylogenetic groups, indicating a conserved functional diversity within these two species. Conversely, Groups I, VIII, XIII, and XIV lacked rice UBP representatives, highlighting evolutionary differences in the functional specialization of UBP genes between soybean and rice.

**Figure 6:**
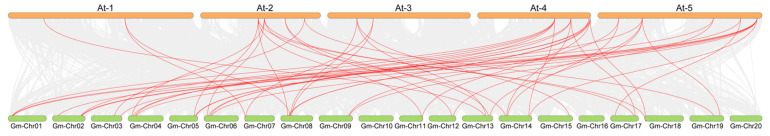

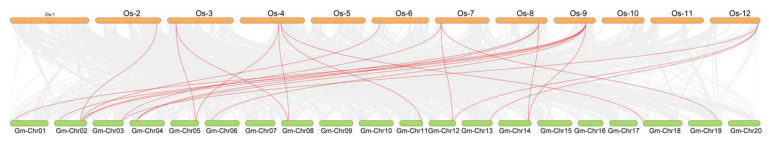
The collinearity analysis of *UBP* genes among Arabidopsis, soybean, and rice. The gray lines depict all gene duplication events during evolution in the genomes of three different species. The red lines illustrate *UBP* gene duplication within Arabidopsis, soybean, and rice. The green columns represent the chromosomes of soybean, while the orange columns correspond to the chromosomes of Arabidopsis and rice.

To elucidate the origin and evolutionary trajectory of the GmUBP gene family, we performed a comprehensive collinearity analysis by generating a collinearity map of *Glycine max* (soybean) against *Arabidopsis thaliana* and *Oryza sativa* (Figure 6). This analysis reveals 51 significant collinearity relationships between soybean and Arabidopsis genes, while only 23 such relationships were identified with rice orthologs. These findings suggest that the GmUBP gene family exhibits a closer evolutionary relationship with Arabidopsis, a conclusion that is strongly supported by phylogenetic tree analysis.

### 2.5. Expression Patterns of UBP Family in Different Tissues

**Figure 7:**
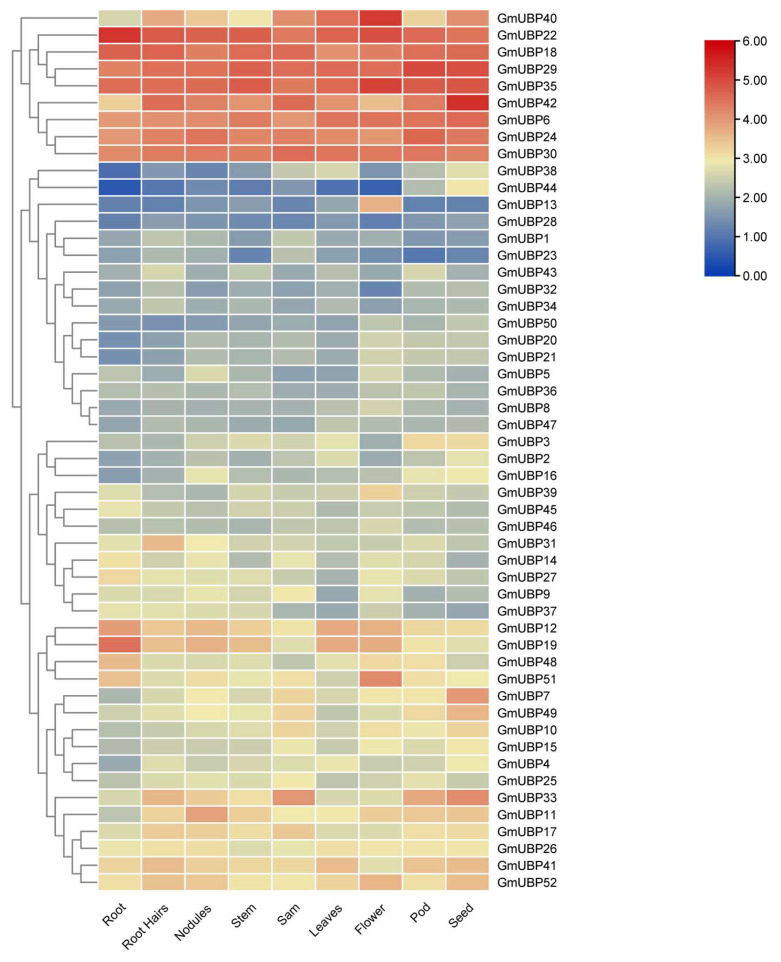
The expression levels of 52 *GmUBP* genes in 9 different tissues of soybean. The expression levels of *GmUBPs* were calculated as log2^FPKM+1^ values and normalized and are displayed in the heat map shown. On the right side of the heat map, a bar box indicates the expression intensity from high to low, with red representing high expression and blue representing low expression.

To further investigate the biological functions of the *UBP* gene family in soybean, we analyzed the expression profiles of *GmUBPs* across various tissues, including the root, root hairs, nodule, stem, shoot apical meristem (SAM), leaves, flower, pod, and seed (Figure 7). The expression patterns of the 52 *GmUBP* genes were categorized into four distinct groups. The first group comprises nine genes, such as *GmUBP40*, which exhibits the highest expression levels across multiple tissues. The second group, consisting of 16 genes (e.g., *GmUBP12* and *GmUBP52*), displays overall high expression levels, despite a relatively low expression in some tissues (Figure 7). Notably, *GmUBP7*, whose high expression in seeds is analogous to that of *AtUBP14* (*AT3G20630*), is suggested to have an important role in seed development or maturation. The third group includes 16 genes (e.g., *GmUBP38* and *GmUBP47*) characterized by low expression across most tissues, except for *GmUBP13*, which shows the highest expression in flowers. Among these, *GmUBP31* is highly expressed in root hairs, while *GmUBP39* is prominently expressed in flowers, indicating potential involvement in soybean flowering. Furthermore, *GmUBP* transcripts in pods and seeds generally demonstrated higher expression levels compared to other tissues, implying that GmUBP-mediated deubiquitination is predominantly utilized to regulate reproductive growth rather than vegetative development.

**Figure 8:**
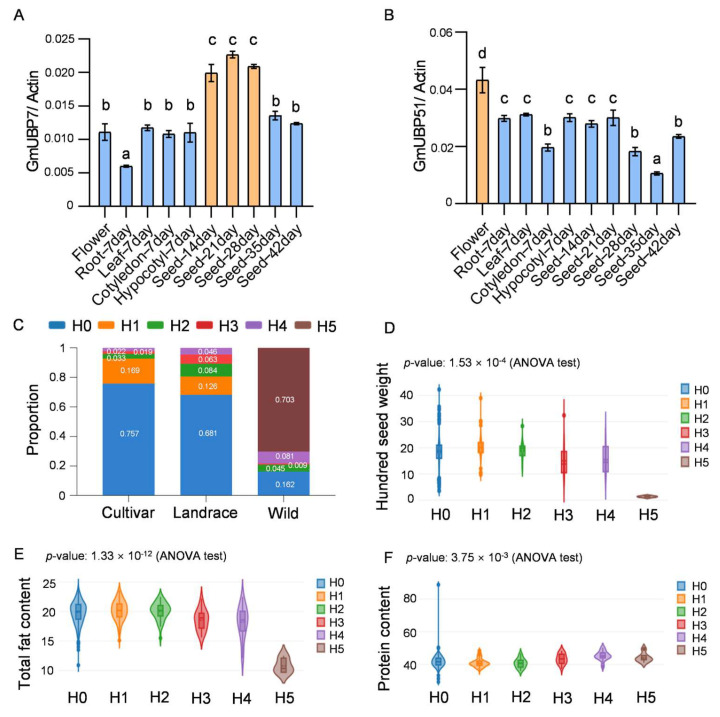
The expression patterns and haplotype analysis of GmUBP genes in soybean. (**A**): The expression of GmUBP7 in different tissues of soybean by qRT−PCR. (**B**): The expression of GmUBP51 in different tissues of soybean by qRT−PCR. The mean expression value was calculated from three independent biological replicates. The statistical method employed was One−way ANOVA test, *p* < 0.05, and a−d letters indicate statistically significant differences. (**C**): The GmUBP7 allele frequency in three sub-populations, including cultivars, landraces, and wild. The five colors used correspond to the five haplotypes, with H0 representing the reference genome (W82), and the rest are alleles in the sub-populations. (**D**): The correlation analysis of five haplotypes and the one-hundred-seed weight of GmUBP7. (**E**): The correlation analysis of five haplotypes and the total fat content of GmUBP7. (**F**): The correlation analysis of five haplotypes and the protein content of GmUBP7. The phenotypic data are from the Soybean Multi-Omics Database, and the statistical method employed was an ANOVA test.

In addition, based on the results of online transcriptome data, we selected two *GmUBP* genes to further determine their expression patterns. *GmUBP7* and *GmUBP51* were expressed in ten tissues (flower and root—7 days, leaf—7 days, cotyledon—7 days, hypocotyl—7 days, seed—14 days, seed—21 days, seed—28 days, seed—35 days, and seed—42 days) with relative expression levels. The results show that the qRT-PCR expression patterns of the two genes were consistent with the general trends of transcriptome expression patterns. *GmUBP7* is highly expressed in seeds, especially in the early and middle stages of seed development, while *GmUPB51* is highly expressed in flowers (Figure 8A,B). However, while *GmUBP51* expression was slightly higher in flowers than in other tissues, it was not as different as the transcriptome data showed, which may have been due to sample differences in sequencing and qRT-PCR.

### 2.6. Functional Analysis of the GmUBP Family Genes

To investigate the potential selection and biological functions of the soybean *GmUBP* gene family, we performed a haplotype analysis using the Soybean Multi-Omics Database. In a comprehensive analysis of 4414 re-sequenced soybean accessions, all 52 *GmUBP* genes exhibited distinct haplotypes. Of these, we found members in each subfamily that demonstrated evidence of selection across wild soybean, landraces, and cultivars. Therefore, we present the haplotypes of 14 members as being representative of the 14 groups. (Figure S1). This observation suggests that most of the *GmUBP* genes have undergone selection during soybean domestication, which may be attributed to either passive selection due to genetic linkage with other genes or active selection driven by advantageous phenotypes.

Furthermore, we speculated on the function of genes based on their specific expression in tissues. As flowering- and seed-related traits are important agrological traits of soybean, we focused on the genes *GmUBP7* and *GmUBP51*, which are highly expressed in flowers and seeds in the transcriptome, and further verified them (Figure 7). In both genes, the qPCR results of *GmUBP7* were consistent with the transcriptome, and both showed that the gene was highly expressed during the early and middle stages of seed development. However, the expression level of *GmUBP51* in flowers was different from that of the transcriptome; therefore, we focused only on *GmUBP7* for further haplotype analysis (Figure 8A,B). The haplotype analysis of this gene reveals notable shifts in the proportions of the H0 and H5 haplotypes across wild soybean, landraces, and cultivars. Specifically, the frequency of H0 increased during domestication, while the frequency of H5 decreased (Figure 8C, Table S8). The phenotypic correlation analysis of the five haplotypes with database phenotypic data demonstrates significant differences in the hundred-seed weight, total fat content, and protein content between H0–H4 and H5 (Figure 8D–F). In addition, H0 also appears to have been subjected to artificial selection, but its seed phenotype was not significantly different from that of H1–H4, probably because H0 is associated with other traits, such as seed coat thickness and germination rate. These findings suggest that *GmUBP7* may have been subjected to selection during domestication. The high expression levels of *GmUBP7* in seeds, as well as its association with seed traits, highlight its potential importance in soybean improvement programs.

## 3. Discussion

As an essential form of protein modification, ubiquitination significantly impacts various cellular processes, such as hormone signaling, stress responses, and organ development [13,23,24,26]. Deubiquitination, serving as a reversible counterpart to ubiquitination, involves the removal of ubiquitin from modified substrates by deubiquitinating enzymes (DUBs), thereby regulating the levels of ubiquitination on target proteins. USPs/UBPs, a major category of DUBs, form a diverse group of enzymes comprising numerous members. Compelling evidence demonstrates that distinct *UBPs* play varied and vital roles in plant development and stress responses [35,36,37,38,39]. The *UBP* gene family has been studied in several species, including *Arabidopsis thaliana* [10], *Oryza sativa* [11], *Phyllostachys edulis* [12], *Triticum aestivum* [13], and *Zea mays* [14]. However, research into the function of *GmUBPs* in soybean development remains underexplored. In this study, we conducted a thorough genome-wide analysis of soybean *UBP* genes utilizing publicly accessible genome data. Additionally, we examined the potential roles of *UBP* genes in soybean by analyzing genetic variation and phenotypic data.

In the present investigation, structural analysis reveals that all characterized GmUBP proteins harbored the UCH domain, which is characterized by the presence of conserved Cys and His-box motifs (Figure 1). Furthermore, a comparison of domain composition among different genetic clades indicates that members within more closely related branches tended to share identical structural motifs, whereas proteins from distinct clades exhibited variations in their domain composition (Figure 1). These findings suggest inherent differences in substrate affinity and functional roles among the *GmUBP* family members. Soybean, as a recognized paleopolyploid genome [40], has undergone a series of whole-genome duplication events, including the Gamma event (or WGT), which is estimated to have occurred prior to the divergence of monocots and dicots approximately 300 million years ago [41]. Additional polyploidization events, such as legume-wide genome duplication (WGD) around 59 million years ago and Glycine-specific WGD approximately 13 million years ago [42,43], have collectively contributed to the highly duplicated nature of the soybean genome. Currently, over 70% of soybean genes exist in multiple copies [44,45]. In the current study, a total of 47 *GmUBP* gene pairs resulting from segmental duplication were identified, with no evidence of tandem duplications (Figure 4). This observation implies that segmental duplication may predominantly drive the expansion of the *GmUBP* gene family.

In this study, all *GmUBP* promoters contain light-responsive elements, which serve as binding sites for transcription factors mediating gene expression in response to light signals (Figure 2). Therefore, light signals may regulate the stability of downstream gene products by influencing the expression of *GmUBP* genes; this is a process that is associated with photoreception [46]. Similarly, approximately two-thirds of the *GmUBP* promoters contain anaerobic-responsive elements (Figure 2). These elements represent specific cis-acting sequences found in the promoter regions of the genes activated under low-oxygen conditions, such as flooding or soil waterlogging. Such elements facilitate the binding of transcription factors, thereby enhancing the plant’s ability to respond to hypoxic or anoxic stress through the activation of genes involved in anaerobic responses [47]. Moreover, the promoters of *GmUBP* genes are characterized by abundant stress-responsive cis-elements, including those associated with low-temperature and drought conditions (Figure 2). This suggests that *GmUBP* genes likely play significant roles in abiotic stress responses. Specifically, when plants detect abiotic stress signals, certain transcription factors may bind to these cis-elements, activating the transcription of *GmUBP* genes. The resulting *GmUBP* proteins then cleave ubiquitin chains from ubiquitinated proteins, thereby regulating multiple physiological pathways to enable the plant to cope with abiotic stresses. Additionally, plant hormones are key signaling molecules that regulate various physiological processes during plant growth and development [47]. Our analysis reveals that the promoter regions of *GmUBP* genes contain multiple hormone-responsive elements, including those responsive to jasmonic acid, abscisic acid, gibberellin, and auxin (Figure 2). This finding suggests that soybean *UBP* genes may actively participate in hormone signaling pathways, potentially integrating hormonal regulation with other stress response mechanisms.

Soybean, a vital legume crop, serves as an excellent source of both protein and oil. Enhancing soybean productivity hinges on elucidating and comprehending the mechanisms underlying the regulation of seed-related traits. Research has demonstrated that various UBP (ubiquitin-specific protease) family members play significant and diverse roles in controlling different traits. However, aside from Arabidopsis *UBP14* [26], rice *OsUBP15* [24], and *GmSW17* (the ortholog of *AtUBP22*) [33], the roles of other *UBPs* in seed development remain largely unexplored. In this study, we identified *GmUBP7*—a homolog of Arabidopsis *UBP5*—which is highly expressed in soybean seeds and potentially regulates seed size and lipid–protein content (Figure 8). *Glycine max*, the cultivated soybean, was domesticated in China approximately 5000–9000 years ago from its wild progenitor, *Glycine soja*. This domestication process is typically marked by an increasing trend in 100-seed weight, accompanied by a decline in protein content and a concomitant rise in oil content [48,49,50]. Our analysis reveals that the frequency of *GmUBP7*-H0 increased throughout the domestication process, while the frequency of H5 decreased. Importantly, the seed phenotypes associated with H0 and H5 aligned with the general trajectory of domestication. These findings suggest that *GmUBP7* may function as a key domestication gene, with *GmUBP7*-H0 playing a pivotal role in the domestication process (Figure 8). Further analysis reveals that H5 exhibited base deletion (CCCAAACAAAGTGATTTA) in the first intron region of the *GmUBP7* gene compared to H0, which could alter the gene’s function (Table S8). It has been suggested that variations in introns may impact the binding capacity of upstream transcription factors, thereby regulating gene expression [51,52]. To investigate whether H5 deletion represented a transcription factor binding site, we utilized the online tool PlantPAN4.0. The analyses indicate that the deleted sequence contains recognition sites for NFY family transcription factors, specifically NF-YA, NF-YB, and NF-YC. It is known that NFY family members are involved in regulating seed size and oil content in soybean and maize [53,54,55,56]. Therefore, *GmUBP7* may collaborate with NFY family members to regulate seed development, and the phenotypic differences observed between H0-4 and H5 might result from varying NFY transcription factor binding affinities to the *GmUBP7* gene.

In conclusion, the bioinformatic analysis of soybean *UBP* genes presented in this study offers a comprehensive overview of their chromosomal localization, motif and domain structures, expression patterns, and haplotype analysis. These findings provide valuable insights and serve as key references for the further characterization and functional studies of specific *GmUBP* genes in soybean.

## 4. Materials and Methods

This section is divided into subheadings. It should provide a concise and precise description of the experimental results and their interpretation, as well as the experimental conclusions that can be drawn.

### 4.1. Identification and Chromosomal Locations of Soybean UBP Genes

We retrieved the protein and genome sequence files for *Glycine max* (Wm82.a2.1) from the plant genome database Phytozome (https://phytozome-next.jgi.doe.gov/, accessed on 3 September 2024), and downloaded the protein and genome sequence files for *Arabidopsis thaliana* and *Oryza sativa* from the EnsemblPlants database (https://plants.ensembl.org/, accessed on 3 September 2024). [57].

To identify *UBP* genes in these species, we used two approaches. First, we extracted the conserved domain UCH (PF00443) from Arabidopsis UBP proteins on PFAM (http://pfam.xfam.org, accessed on 3 September 2024) and utilized TBtools’ Advanced Hmmer Search plugin to search for candidate UBP proteins in soybean, with default parameters. Second, we used Arabidopsis UBP proteins as queries against soybean protein sequences in MEGA11.0 with default settings. Proteins lacking the UCH domain or its characteristic Cys-box and His-box residues were excluded.

The genomic distribution of *UBP* genes was determined using the respective genome and annotation files. Subcellular localization was predicted via the BUSCA server (http://busca.biocomp.unibo.it/, accessed on 9 September 2024) [58]. Physicochemical properties, including amino acid count, molecular weight, and isoelectric point, were analyzed using Tbtools. To further understand their structural features, we predicted transmembrane domains using TMHMM (https://services.healthtech.dtu.dk/service.php?TMHMM-2.0, accessed on 8 September 2024).

### 4.2. Chromosome Mapping and Phylogenetic Analysis

To determine the genomic positions of all identified *UBP* genes in soybean, their chromosomal locations were visualized using TBtools (v2.310). Full-length amino acid sequences from *Glycine max*, *Arabidopsis thaliana*, and *Oryza sativa* were aligned using MEGA11.0 with the MUSCLE function. The phylogenetic tree was constructed by subjecting the multiple sequence alignment file to MEGA11, employing the neighbor-joining (NJ) method with 1000 bootstrap replicates, pairwise deletion, and a Poisson model. Finally, the tree was refined using iTOL (https://itol.embl.de/upload.cgi, accessed on 9 January 2025).

### 4.3. Gene Structure and Conserved Motif Analysis

To examine the gene structure of *UBP* genes in soybean, TBtools was employed for analysis. The conserved structural motifs of *UBP* genes were identified using MEME (https://meme-suite.org/meme/tools/meme, accessed on 24 September 2024) [59]. The results from both gene structure and motif analyses were consolidated using TBtools for visualization.

### 4.4. Analysis of cis-Regulatory Elements of UBP Genes

To examine the upstream regulatory regions of *UBP* genes in soybean, the genomic sequences spanning 2000 bp upstream of the translation initiation site for each *UBP* gene were extracted from the genome files. The cis-acting elements within the *UBP* gene promoters were identified using PlantCare Serve (http://bioinformatics.psb.ugent.be/webtools/plantcare/html/, accessed on 22 September 2024) [60]. Finally, the results of promoter analysis were visualized using TBtools’ Basic Biosequence View function.

### 4.5. Collinearity Analysis

The Dual-Synteny Plot program in TBtools was used to analyze the homology of *UBP* genes across maize and other species, including Arabidopsis, soybean, and rice. To further examine collinearity, we implemented the One-Step MCScanX tool from TBtools [61,62]. The resulting data were visualized using TBtools to provide a comprehensive overview of the homology relationships among these species.

### 4.6. Expression Analysis

SoyBase (http://www.soybase.org, accessed on 29 September 2024) served as a platform for extracting *GmUBP* expression profile data across diverse tissues and developmental stages. Heatmap visualizations of the expression profiles were generated using TBtools. For qRT-PCR, we first planted soybean (W82) in a short-day (a light–dark ratio of 10 h/14 h) greenhouse and sampled tissues at different stages of plant development. Total RNA was extracted from different samples using an Eastep^®^ Super Total RNA Extraction Kit (Promega, Shanghai, China), and cDNA was synthesized from the RNA by a reverse transcription reagent kit (HiScript II 1st Strand cDNA Synthesis Kit (Vazyme, Nanjing, China). Real-time quantitative PCR was performed using 2 × ChamQ Universal SYBR qPCR Master Mix (Vazyme, Nanjing, China). *GmActin* was used as an internal control gene. The gene-specific primers are listed in Table S9.

### 4.7. Haplotype Analysis

The Soybean Multi-omics Database (https://yanglab.hzau.edu.cn/SoyMD/#/, accessed on 13 February 2025) was employed to investigate the haplotype structure of *GmUBP* genes in soybean. During the analysis, the single-locus model was selected to evaluate the gene haplotypes. By inputting the corresponding gene identifier, researchers can retrieve allele frequency data specific to sub-populations. Furthermore, the platform enables the integration of existing phenotypic records with haplotype information, facilitating statistical analysis to determine significant differences between haplotypes.

## 5. Conclusions

In this study, we conducted a comprehensive investigation into the *GmUBP* gene family. Initially, phylogenetic analysis was employed to classify the 52 identified *GmUBP* genes into 14 distinct groups based on their evolutionary relationships with UBP genes from Arabidopsis, rice, and soybean. Subsequent analyses, including exon–intron structure characterization, the identification of conserved domains, and motif analysis, provide robust confirmation of the high conservation of *GmUBP* genes within the same group. Synteny analysis was then employed to elucidate the evolutionary trajectory of the *GmUBP* gene family. Furthermore, promoter cis-regulatory element analysis, combined with tissue-specific expression profiling in *Glycine max*, reveals the potential involvement of *GmUBP* genes in key aspects of plant growth and development. Notably, we identified that *GmUBP7* may have undergone selection during domestication and appears to play a role in the coordinated regulation of seed size and quality. Our findings offer a valuable foundation for future research on the *GmUBP* gene family, particularly with respect to soybean seed development, and provide a scientific framework for exploring the biological functions and mechanisms underlying *GmUBP* gene activity.

## Data Availability

All data generated or analyzed in this study are included in the main text and its Supplementary Materials.

## Supplementary Materials

The following supporting information can be downloaded at: https://www.mdpi.com/article/10.3390/ijms26146689/s1.

## References

[1] Graham P.H., Vance C.P. (2003). Legumes: Importance and constraints to greater use. Plant Physiol..

[2] Li N., Li Y. (2016). Signaling pathways of seed size control in plants. Curr. Opin. Plant Biol..

[3] Zhou H., Zhao J., Cai J., Patil S.B. (2017). UBIQUITIN-SPECIFIC PROTEASES function in plant development and stress responses. Plant Mol. Biol..

[4] Frappier L., Verrijzer C.P. (2011). Gene expression control by protein deubiquitinases. Curr. Opin. Genet. Dev..

[5] Zhang Y. (2003). Transcriptional regulation by histone ubiquitination and deubiquitination. Genes Dev..

[6] Atanassov B.S., Koutelou E., Dent S.Y. (2011). The role of deubiquitinating enzymes in chromatin regulation. FEBS Lett..

[7] Neutzner M., Neutzner A. (2012). Enzymes of ubiquitination and deubiquitination. Essays Biochem..

[8] Amerik A.Y., Hochstrasser M. (2004). Mechanism and function of deubiquitinating enzymes. Biochim. Biophys. Acta.

[9] Wu R., Zheng W., Tan J., Sammer R., Du L., Lu C. (2019). Protein partners of plant ubiquitin-specific proteases (UBPs). Plant Physiol. Biochem..

[10] Yan N., Doelling J.H., Falbel T.G., Durski A.M., Vierstra R.D. (2000). The ubiquitin-specific protease family from Arabidopsis. AtUBP1 and 2 are required for the resistance to the amino acid analog canavanine. Plant Physiol..

[11] Wang D.H., Song W., Wei S.W., Zheng Y.F., Chen Z.S., Han J.D., Zhang H.T., Luo J.C., Qin Y.M., Xu Z.H. (2018). Characterization of the Ubiquitin C-Terminal Hydrolase and Ubiquitin-Specific Protease Families in Rice (*Oryza sativa*). Front. Plant Sci..

[12] Wu R., Shi Y., Zhang Q., Zheng W., Chen S., Du L., Lu C. (2019). Genome-Wide Identification and Characterization of the UBP Gene Family in Moso Bamboo (*Phyllostachys edulis*). Int. J. Mol. Sci..

[13] Xu M., Jin P., Liu T., Gao S., Zhang T., Zhang F., Han X., He L., Chen J., Yang J. (2021). Genome-wide identification and characterization of UBP gene family in wheat (*Triticum aestivum* L.). PeerJ.

[14] Fu W., Fan D., Liu S., Bu Y. (2024). Genome-wide identification and expression analysis of Ubiquitin-specific protease gene family in maize (*Zea mays* L.). BMC Plant Biol..

[15] Zhao J., Zhou H., Zhang M., Gao Y., Li L., Gao Y., Li M., Yang Y., Guo Y., Li X. (2016). Ubiquitin-specific protease 24 negatively regulates abscisic acid signalling in *Arabidopsis thaliana*. Plant Cell Environ..

[16] Liu G., Liang J., Lou L., Tian M., Zhang X., Liu L., Zhao Q., Xia R., Wu Y., Xie Q. (2022). The deubiquitinases UBP12 and UBP13 integrate with the E3 ubiquitin ligase XBAT35.2 to modulate VPS23A stability in ABA signaling. Sci. Adv..

[17] Zhou Y., Park S.H., Chua N.H. (2023). UBP12/UBP13-mediated deubiquitination of salicylic acid receptor NPR3 suppresses plant immunity. Mol. Plant.

[18] Zhou H., Zhao J., Yang Y., Chen C., Liu Y., Jin X., Chen L., Li X., Deng X.W., Schumaker K.S. (2012). Ubiquitin-specific protease16 modulates salt tolerance in Arabidopsis by regulating Na(+)/H(+) antiport activity and serine hydroxymethyltransferase stability. Plant Cell.

[19] Jiang R., Zhou S., Da X., Chen T., Xu J., Yan P., Mo X. (2022). Ubiquitin-Specific Protease 2 (OsUBP2) Negatively Regulates Cell Death and Disease Resistance in Rice. Plants.

[20] Park S.H., Jeong J.S., Seo J.S., Park B.S., Chua N.H. (2019). Arabidopsis ubiquitin-specific proteases UBP12 and UBP13 shape ORE1 levels during leaf senescence induced by nitrogen deficiency. New Phytol..

[21] An Z., Liu Y., Ou Y., Li J., Zhang B., Sun D., Sun Y., Tang W. (2018). Regulation of the stability of RGF1 receptor by the ubiquitin-specific proteases UBP12/UBP13 is critical for root meristem maintenance. Proc. Natl. Acad. Sci. USA.

[22] Cui X., Lu F., Li Y., Xue Y., Kang Y., Zhang S., Qiu Q., Cui X., Zheng S., Liu B. (2013). Ubiquitin-specific proteases UBP12 and UBP13 act in circadian clock and photoperiodic flowering regulation in Arabidopsis. Plant Physiol..

[23] Du L., Li N., Chen L., Xu Y., Li Y., Zhang Y., Li C., Li Y. (2014). The ubiquitin receptor DA1 regulates seed and organ size by modulating the stability of the ubiquitin-specific protease UBP15/SOD2 in Arabidopsis. Plant Cell.

[24] Shi C., Ren Y., Liu L., Wang F., Zhang H., Tian P., Pan T., Wang Y., Jing R., Liu T. (2019). Ubiquitin Specific Protease 15 Has an Important Role in Regulating Grain Width and Size in Rice. Plant Physiol..

[25] Doelling J.H., Phillips A.R., Soyler-Ogretim G., Wise J., Chandler J., Callis J., Otegui M.S., Vierstra R.D. (2007). The ubiquitin-specific protease subfamily UBP3/UBP4 is essential for pollen development and transmission in Arabidopsis. Plant Physiol..

[26] Doelling J.H., Yan N., Kurepa J., Walker J., Vierstra R.D. (2001). The ubiquitin-specific protease UBP14 is essential for early embryo development in *Arabidopsis thaliana*. Plant J..

[27] Luo M., Luo M.Z., Buzas D., Finnegan J., Helliwell C., Dennis E.S., Peacock W.J., Chaudhury A. (2008). UBIQUITIN-SPECIFIC PROTEASE 26 is required for seed development and the repression of PHERES1 in Arabidopsis. Genetics.

[28] Sridhar V.V., Kapoor A., Zhang K., Zhu J., Zhou T., Hasegawa P.M., Bressan R.A., Zhu J.K. (2007). Control of DNA methylation and heterochromatic silencing by histone H2B deubiquitination. Nature.

[29] Zou T., Li G., Liu M., Liu R., Yang S., Wang K., Lu L., Ye Q., Liu J., Liang J. (2023). A ubiquitin-specific protease functions in regulating cell death and immune responses in rice. Plant Cell Environ..

[30] Sun J., Song W., Chang Y., Wang Y., Lu T., Zhang Z. (2021). OsLMP1, Encoding a Deubiquitinase, Regulates the Immune Response in Rice. Front. Plant Sci..

[31] Moon Y.K., Hong J.P., Cho Y.C., Yang S.J., An G., Kim W.T. (2009). Structure and expression of OsUBP6, an ubiquitin-specific protease 6 homolog in rice (*Oryza sativa* L.). Mol. Cells.

[32] Kong J., Jin J., Dong Q., Qiu J., Li Y., Yang Y., Shi Y., Si W., Gu L., Yang F. (2019). Maize factors ZmUBP15, ZmUBP16 and ZmUBP19 play important roles for plants to tolerance the cadmium stress and salt stress. Plant Sci..

[33] Liang S., Duan Z., He X., Yang X., Yuan Y., Liang Q., Pan Y., Zhou G., Zhang M., Liu S. (2024). Publisher Correction: Natural variation in GmSW17 controls seed size in soybean. Nat. Commun..

[34] Wang P., Moore B.M., Panchy N.L., Meng F., Lehti-Shiu M.D., Shiu S.H. (2018). Factors Influencing Gene Family Size Variation Among Related Species in a Plant Family, Solanaceae. Genome Biol. Evol..

[35] Zheng L., Wu H., Qanmber G., Ali F., Wang L., Liu Z., Yu D., Wang Q., Xu A., Yang Z. (2020). Genome-Wide Study of the GATL Gene Family in *Gossypium hirsutum* L. Reveals that *GhGATL* Genes Act on Pectin Synthesis to Regulate Plant Growth and Fiber Elongation. Genes.

[36] Liu Y., Wang F., Zhang H., He H., Ma L., Deng X.W. (2008). Functional characterization of the Arabidopsis ubiquitin-specific protease gene family reveals specific role and redundancy of individual members in development. Plant J..

[37] March E., Farrona S. (2017). Plant Deubiquitinases and Their Role in the Control of Gene Expression Through Modification of Histones. Front. Plant Sci..

[38] Ewan R., Pangestuti R., Thornber S., Craig A., Carr C., O’Donnell L., Zhang C., Sadanandom A. (2011). Deubiquitinating enzymes AtUBP12 and AtUBP13 and their tobacco homologue NtUBP12 are negative regulators of plant immunity. New Phytol..

[39] Godwin J., Govindasamy M., Nedounsejian K., March E., Halton R., Bourbousse C., Wolff L., Fort A., Krzyszton M., López Corrales J. (2024). The UBP5 histone H2A deubiquitinase counteracts PRCs-mediated repression to regulate Arabidopsis development. Nat. Commun..

[40] Walling J.G., Shoemaker R., Young N., Mudge J., Jackson S. (2006). Chromosome-level homeology in paleopolyploid soybean (*Glycine max*) revealed through integration of genetic and chromosome maps. Genetics.

[41] Bowers J.E., Chapman B.A., Rong J., Paterson A.H. (2003). Unravelling angiosperm genome evolution by phylogenetic analysis of chromosomal duplication events. Nature.

[42] Severin A.J., Cannon S.B., Graham M.M., Grant D., Shoemaker R.C. (2011). Changes in twelve homoeologous genomic regions in soybean following three rounds of polyploidy. Plant Cell.

[43] Cannon S.B., Shoemaker R.C. (2012). Evolutionary and comparative analyses of the soybean genome. Breed. Sci..

[44] Schmutz J., Cannon S.B., Schlueter J., Ma J., Mitros T., Nelson W., Hyten D.L., Song Q., Thelen J.J., Cheng J. (2010). Genome sequence of the palaeopolyploid soybean. Nature.

[45] Wang W., Jiang W., Liu J., Li Y., Gai J., Li Y. (2017). Genome-wide characterization of the aldehyde dehydrogenase gene superfamily in soybean and its potential role in drought stress response. BMC Genom..

[46] Casal J.J., Yanovsky M.J. (2005). Regulation of gene expression by light. Int. J. Dev. Biol..

[47] Mohanty B., Krishnan S.P., Swarup S., Bajic V.B. (2005). Detection and preliminary analysis of motifs in promoters of anaerobically induced genes of different plant species. Ann. Bot..

[48] Liu Y., Du H., Li P., Shen Y., Peng H., Liu S., Zhou G.A., Zhang H., Liu Z., Shi M. (2020). Pan-Genome of Wild and Cultivated Soybeans. Cell.

[49] Sedivy E.J., Wu F., Hanzawa Y. (2017). Soybean domestication: The origin, genetic architecture and molecular bases. New Phytol..

[50] Zhou Z., Jiang Y., Wang Z., Gou Z., Lyu J., Li W., Yu Y., Shu L., Zhao Y., Ma Y. (2016). Erratum: Resequencing 302 wild and cultivated accessions identifies genes related to domestication and improvement in soybean. Nat. Biotechnol..

[51] Aguilar-Hernández V., Guzmán P. (2013). Spliceosomal introns in the 5′ untranslated region of plant BTL RING-H2 ubiquitin ligases are evolutionary conserved and required for gene expression. BMC Plant Biol..

[52] Rose A.B., Carter A., Korf I., Kojima N. (2016). Intron sequences that stimulate gene expression in Arabidopsis. Plant Mol. Biol..

[53] Zhang D., Zhao M., Li S., Sun L., Wang W., Cai C., Dierking E.C., Ma J. (2017). Plasticity and innovation of regulatory mechanisms underlying seed oil content mediated by duplicated genes in the palaeopolyploid soybean. Plant J..

[54] Lu L., Wei W., Li Q.T., Bian X.H., Lu X., Hu Y., Cheng T., Wang Z.Y., Jin M., Tao J.J. (2021). A transcriptional regulatory module controls lipid accumulation in soybean. New Phytol..

[55] O’Conner S., Zheng W., Qi M., Kandel Y., Fuller R., Whitham S.A., Li L. (2021). GmNF-YC4-2 Increases Protein, Exhibits Broad Disease Resistance and Expedites Maturity in Soybean. Int. J. Mol. Sci..

[56] Zhang M., Zheng H., Jin L., Xing L., Zou J., Zhang L., Liu C., Chu J., Xu M., Wang L. (2022). miR169o and ZmNF-YA13 act in concert to coordinate the expression of ZmYUC1 that determines seed size and weight in maize kernels. New Phytol..

[57] Goodstein D.M., Shu S., Howson R., Neupane R., Hayes R.D., Fazo J., Mitros T., Dirks W., Hellsten U., Putnam N. (2012). Phytozome: A comparative platform for green plant genomics. Nucleic Acids Res..

[58] Savojardo C., Martelli P.L., Fariselli P., Profiti G., Casadio R. (2018). BUSCA: An integrative web server to predict subcellular localization of proteins. Nucleic Acids Res..

[59] Bailey T.L., Johnson J., Grant C.E., Noble W.S. (2015). The MEME Suite. Nucleic Acids Res..

[60] Lescot M., Déhais P., Thijs G., Marchal K., Moreau Y., Van de Peer Y., Rouzé P., Rombauts S. (2002). PlantCARE, a database of plant cis-acting regulatory elements and a portal to tools for in silico analysis of promoter sequences. Nucleic Acids Res..

[61] Chen C., Wu Y., Li J., Wang X., Zeng Z., Xu J., Liu Y., Feng J., Chen H., He Y. (2023). TBtools-II: A “one for all, all for one” bioinformatics platform for biological big-data mining. Mol. Plant.

[62] Chen C., Chen H., Zhang Y., Thomas H.R., Frank M.H., He Y., Xia R. (2020). TBtools: An Integrative Toolkit Developed for Interactive Analyses of Big Biological Data. Mol. Plant.

