# Supplementary material for: Genome-Wide Identification and Comprehensive Analysis of Ubiquitin-Specific Protease Gene Family in Soybean (Glycine max)
Source: Int J Mol Sci. 2025 Jul 11;26(14):6689. doi: 10.3390/ijms26146689 (PMC12294657; doi:10.3390/ijms26146689)
Supplement: Supplementary file 1 [file ijms-26-06689-s001.zip › Supplementary figure.pdf]

Genome-Wide Identification and Comprehensive Analysis of Ubiquitin-Specific Protease Gene Family in Soybean (*Glycine max*)

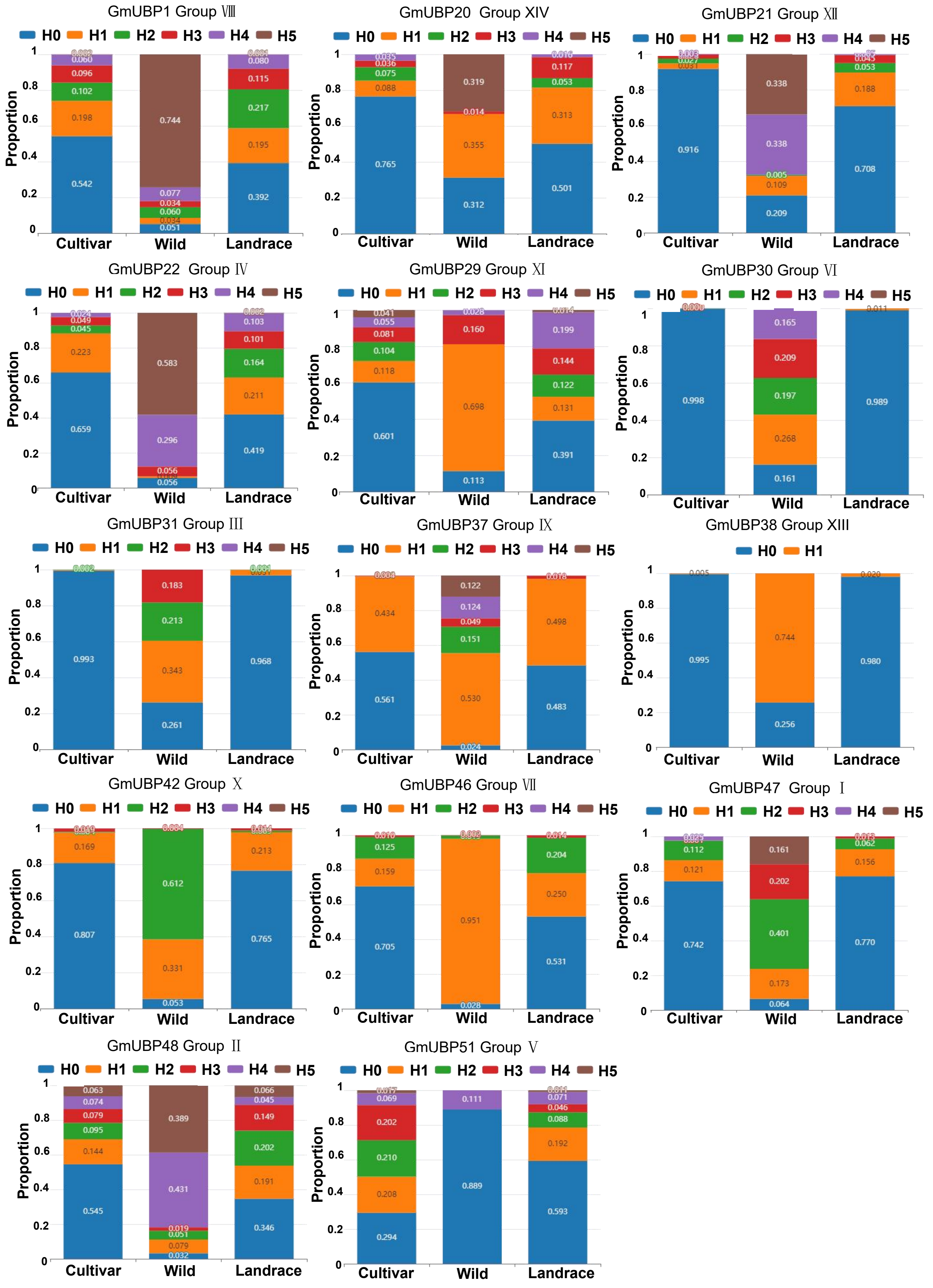

Figure S1. Haplotype frequency of *GmUBP* genes in soybean. This picture shows the allele frequency in three sub-population of 14 *GmUBP* family members, including cultivar, landrace and wild. The five colors used correspond to the five haplotypes, with H0 representing the reference genome (W82), and the rest are allele in sub-population. The data are from the Soybean multi-omics database.
